# Improving surveillance of infectious diseases in child daycare settings: insights from a pilot study in the Netherlands

**DOI:** 10.3389/fpubh.2026.1797334

**Published:** 2026-04-10

**Authors:** Rosaline van den Berg, Elisabeth Huijskens, Fleur Groenendijk, Gregorius J. Sips, Hélène Voeten, Aimée Tjon-A-Tsien, Joan Roozemond, Rémy Schilperoort, Gertjan Medema, Raïssa Tjon-Kon-Fat

**Affiliations:** 1 Department of Infectious Disease Control, Public Health Service Zuid-Holland Zuid, Dordrecht, Netherlands; 2 Department of Medical Microbiology, Albert Schweitzer Hospital, Dordrecht, Netherlands; 3 Department of Infectious Disease Control, Public Health Service Zeeland, Goes, Netherlands; 4 Public Health Service Rotterdam-Rijnmond, Rotterdam, Netherlands; 5 Department of Public Health, Erasmus MC, Rotterdam, Netherlands; 6 Partners4UrbanWaters, Nijmegen, Netherlands; 7 KWR Water Research Institute, Nieuwegein, Netherlands

**Keywords:** child daycare centers, communicable diseases, feces sampling, incidence, syndromic surveillance, wastewater-based epidemiological monitoring

## Abstract

**Introduction:**

Child daycare centres (DCCs) are high-risk settings for infectious disease transmission. Although Dutch DCCs are legally required to report unusually high numbers of infections to Public Health Services (PHS), underreporting is common. Our pilot study aims to enhance DCC surveillance using a simple paper-based syndromic tool and wastewater monitoring.

**Methods:**

Large DCCs in the Dutch PHS-region Zuid-Holland Zuid were invited. During 16 weeks in summer and winter, DCCs recorded weekly counts of children (0–4 years) and staff with respiratory, gastrointestinal and skin infections using tally sheets. Incidence rates/100 persons/week were calculated and compared between seasons (Wilcoxon signed-rank test; DCC-level). In parallel, feasibility of local wastewater surveillance was assessed in stand-alone DCCs through sewer map review, on-site inspection and dye testing to identify DCC-specific sampling points, with planned analysis for parvovirus B19, norovirus and respiratory syncytial virus.

**Results:**

Twenty-six DCCs participated in summer and 23 in winter. DCCs reported 7 mandatory outbreak notifications in summer and 4 in winter. Tally sheets showed a higher incidence in winter than in summer (20.1 vs. 8.8/100 persons/week; *p* < 0.001). Respiratory infections predominated in summer and winter. Ten DCCs were assessed for wastewater surveillance feasibility. Due to infrastructural constraints, no DCC was suitable and wastewater surveillance was not performed.

**Conclusion:**

In our pilot study, paper-based syndromic surveillance provided insights into infectious disease incidences in DCCs beyond mandatory notifications, but was perceived as burdensome, whereas wastewater surveillance proved infeasible. Future DCCs surveillance should prioritize low-burden, scalable systems independent of healthcare-seeking, testing and reporting behaviour.

## 1 Introduction

Infectious diseases can spread rapidly, particularly in settings where groups gather for one or more parts of the day. This is especially true for young children due to their immature immune systems and limited awareness and practice of hygiene measures (1). The risk is further exacerbated by the high child density in confined childcare settings (2). Young children also may shed pathogens for extended periods following infection, potentially including strains with antimicrobial resistance, which can lead to an increased risk of transmission to their environment. Moreover, hand hygiene compliance among caregivers at child daycare centers (DCCs) is often suboptimal, potentially facilitating further transmission (3). Consequently, infections may spread into the wider community, contributing to increased disease burden, healthcare utilization, and parental work absenteeism (1).

To enable early detection and prompt public health responses to acute infectious disease threats in DCCs, a mandatory reporting system is in place in the Netherlands. DCCs are required to report unusual numbers of children or caregivers presenting with symptoms to the Public Health Service (PHS) of their region (4). Reportable symptoms include: diarrhea, jaundice, skin conditions, or other serious conditions suspected to be of infectious origin.

Despite this mandatory system, several studies have shown low outbreak reporting rates (5–7). Between 2015 and 2021, 45–80% of the DCCs in the South-Western region of the Netherlands had never reported an outbreak, despite strong indications that DCCs experience multiple outbreaks each year (8). DCCs often refrain from contacting the PHS in case of minor, recurring, or limited outbreaks, but typically do so when the illness is severe, widespread, persistent, or when it generates uncertainty or concern within the DCC. Non-reporting is thus often unintentional (1, 7).

In addition to the underreporting of outbreaks, limited diagnostic testing poses a significant challenge. Diagnostic testing is often not conducted, as obtaining invasive samples from young children can be difficult and time consuming. Consequently, insight into the circulation of infectious diseases in young children remains limited. This lack of data is challenging, as understanding infection dynamics is crucial for guiding targeted control measures and monitoring (emerging) pathogens.

Despite challenges, DCCs represent a particularly suitable setting for routine surveillance among young children. In the Netherlands, around 60% of children aged 0–4 years attend a DCC, which typically operates year-round (9). Absenteeism in childcare is almost always directly linked to illness. Although not legally mandated, absenteeism is routinely recorded in DCCs as part of health and safety policies aimed at supporting monitoring and ensuring continuity of care (10).

Our pilot study aims to enhance infectious disease surveillance in DCCs through the use of a simple paper-based tool to systematically record infectious disease symptoms (tally sheets) and wastewater monitoring, and to examine how the paper-based system and the wastewater monitoring relate to each other. These methods will lead to more insight into the occurrence of infectious diseases in DCCs, not only during outbreaks but also under non-outbreak conditions—referred to as background prevalence. Ultimately, this approach is intended to improve the early detection of infection trends in DCCs, enabling earlier public health measures to contain (potential) outbreaks.

This study was conducted in the South-Western region of the Netherlands (PHS region Zuid-Holland Zuid), comprising 10 municipalities that together reflect a mix of urban and rural settings, industrial activity, agriculture, and a National Park (the Biesbosch). It has a diverse socioeconomic profile, a relatively low vaccination coverage compared to the Dutch national average, and specific environmental health challenges related to industry and shipping. About 2.6% of the Dutch population lives in this region, and the proportion of 0–4-year-olds is comparable to the national average.

## 2 Methods

### 2.1 Study design

This is a prospective dynamic cohort study, executed during the summer and winter of 2024. The study consisted of three pillars: pillar 1) active surveillance via tally sheets aiming to gain insight into the occurrence of symptoms of infectious diseases; pillar 2) wastewater surveillance to monitor the circulation of pathogens in wastewater; and pillar 3) exploring the relation between pillar 1 and 2, i.e., pathogen detection in wastewater and observed disease occurrence.

### 2.2 Study population

Recruitment of DCCs took place in April 2024. We obtained a list of all DCCs in the South-Western PHS region of the Netherlands (PHS Zuid-Holland Zuid; ~470.000 population) from the Dutch National Childcare Register (11). This register is continuously updated and contains only childcare facilities that have been checked and approved by the municipalities and the PHS. In March 2024, the register contained 248 DCCs in this region that provided full time daycare for children aged 0–4.

We excluded DCCs falling below the 75th percentile in terms of child enrollment. This cut-off was chosen because larger DCCs potentially offer more opportunities for pathogen introduction and exposure (12), and to ensure the anonymity of participating children and staff. In total, 60 DCCs fell above the 75th percentile in terms of child enrollment and those 60 DCCs accounted for 51.5% of the total DCC children’s capacity in the region.

The 60 DCCs were informed about the study and voluntarily decided whether to participate. Children and staff within participating centres were automatically included without further inclusion/exclusion criteria (pillar 1). For pillar 2, additional eligibility criteria applied. Only DCCs that were not part of an integrated child centre or co-located with an after-school care facility were eligible. DCCs located within apartment complexes or nursing homes were also excluded. Based on these criteria, 16 of the 60 DCCs were eligible to participate in wastewater surveillance (pillar 2). Logically, diaper-wearing children attending eligible DCCs did not contribute to pillar 2, as they do not use sanitary facilities and thus do not directly contribute to the wastewater stream. Consequently, the same eligibility criteria applied to pillar 3 by default.

### 2.3 Data collection

Baseline characteristics of participating DCCs, such as capacity and group structure, were collected. Degree of urbanization and socioeconomic status (SES) of the area where the DCC was located was based on the four-digit postal code area. For SES, we used the area-level composite SES-WOA index, which integrates three key characteristics of the local population: (1) financial wellbeing (based on income and assets) [W], (2) educational level [O], and (3) employment continuity over the preceding 4 years [A] (13).

#### 2.3.1 Pillar 1: active surveillance using tally sheets

Participating DCCs were asked to record new illness episodes among children and staff on a weekly basis. Each week, DCCs registered every child or staff member who developed one or more predefined symptoms (Table 1), either occurring at the DCC or reported by parents/caregivers. These predefined symptoms were chosen because they are easy to recognize and are aligned with the national daycare guidelines for infectious diseases as described in the KIDDI app (14), and correspond to the mandatory reporting system. No additional training was provided, as childcare professionals are legally required to recognize these symptoms as part of their reporting responsibilities. A free text field allowed DCCs to report additional symptoms not covered by the predefined categories.

**Table 1:** Predefined illness symptoms based on descriptions in the KIDDI app (https://kiddi.rivm.nl/).

Gastrointestinal symptoms	>3 episodes of watery diarrhea/day, frequent abdominal pain, and/or vomiting
Skin rash	Spots, pustules, and/or ulcers suspected to be of infectious origin
Scabies	Diagnosis confirmed by a physician
Jaundice	Yellow discoloration of sclerae, skin, and/or mucous membranes, with dark urine and pale stools
Respiratory symptoms	Frequent sneezing and/or coughing, persistent nasal discharge
Other severe conditions	e.g. meningitis or multiple pneumonia cases within a short period
Reported ill	Cause unknown

Weekly, aggregated anonymous counts of new illness episodes (on group level) were reported to the research team. Illness episodes were stratified by diaper-wearing and non-diaper-wearing children; staff and non-diaper-wearing children were not registered separately. No personal or medical information was collected or shared with the research team.

To define the weekly population at risk, DCCs also reported the number of attending children and staff present each week, as attendance may fluctuate. All reports were checked for completeness by a researcher. If reports were incomplete or unclear, DCCs were contacted for clarification or to provide missing information. In addition, DCCs received regular follow-up by phone or email to support optimal surveillance response.

Data collection took place during two 8-week periods: summer (week 21–28, 2024) and winter (week 43–50, 2024). The summer period was scheduled to avoid major holidays and school vacations, while the winter period started around the onset of the influenza season. Including both periods allowed for comparison of infectious disease incidence across seasons.

#### 2.3.2 Pillar 2: wastewater surveillance

For each DCC, the possibilities for wastewater monitoring were studied. The objective was to find a monitoring location at which all wastewater from a DCC could be monitored, but without any other (upstream) wastewater sources. Maps of the public sewer system were obtained from the respective municipalities and were used to identify probable discharge locations to the sewer. Dye testing was used to confirm these discharge locations in the field (i.e., discharging dye into, e.g., a toilet of the DCC and observing the dye at a potential monitoring location in a sewer manhole). Also, the field visits were used to assess accessibility to the sewer as well as safe working conditions around the sewer. Wastewater surveillance, would be performed using passive samplers that would remain in place for 48–72 h—if feasible (15, 16).

Laboratory analysis of the samples for parvovirus B19, norovirus and respiratory syncytial virus performed by KWR was planned. The selection of parvovirus B19, norovirus, and RSV was based on their epidemiological relevance in young children, prior evidence of detectability in wastewater, and laboratory feasibility within the available project scope. Given financial and logistical constraints, comprehensive multi-pathogen testing was not feasible within this pilot study. We intended to perform quantitative (RT-)q or droplet digital (dd)PCR and report results as concentrations (or below detection limit) for qPCR, and as concentrations with the number of positive droplets and total droplets (or below detection limit) for ddPCR.

#### 2.3.3 Pillar 3: integrated analysis

We intended to compare data from pillar 1 to pillar 2. In addition, publicly available regional SARS-CoV-2 wastewater data from the Dutch National Wastewater Surveillance by the Dutch National Institute for Public Health and the Environment (RIVM) were descriptively reviewed to contextualize syndromic trends observed in DCCs (17).

### 2.4 Statistical analyses

Descriptive statistics were used to describe the DCCs and their populations. Median and interquartile range (IQR) of children and staff who presented with symptoms per week were described, for summer and winter. Weekly incidence was estimated as the number of children and staff presenting with symptoms divided by the total number of children and staff at risk per week, for every predefined illness. Incidence rates per 100 persons/week were compared between seasons using the Wilcoxon signed rank test, on DCC level. Given the relatively small sample size and the likelihood of non-normally distributed incidence data, a non-parametric approach was used. Although the composition of children attending each DCC varied between seasons, the DCC itself remained a stable analytical unit over time. Therefore, seasonal comparisons were conducted at daycare-centre level, treating each centre as its own control. Results were also compared to mandatory infectious disease notifications from the participating DCCs reported to the PHS. To provide contextual insight into routine reporting practices, the proportion of DCCs submitting at least one mandatory notification under the Dutch Public Health Act during the study periods was compared between participating and non-participating DCCs. For non-participating DCCs, only routine notification data were available. Given the large and heterogeneous catchment areas of the wastewater treatment plants (WWTPs), no formal correlation analysis was performed.

## 3 Results

**Figure 1:**
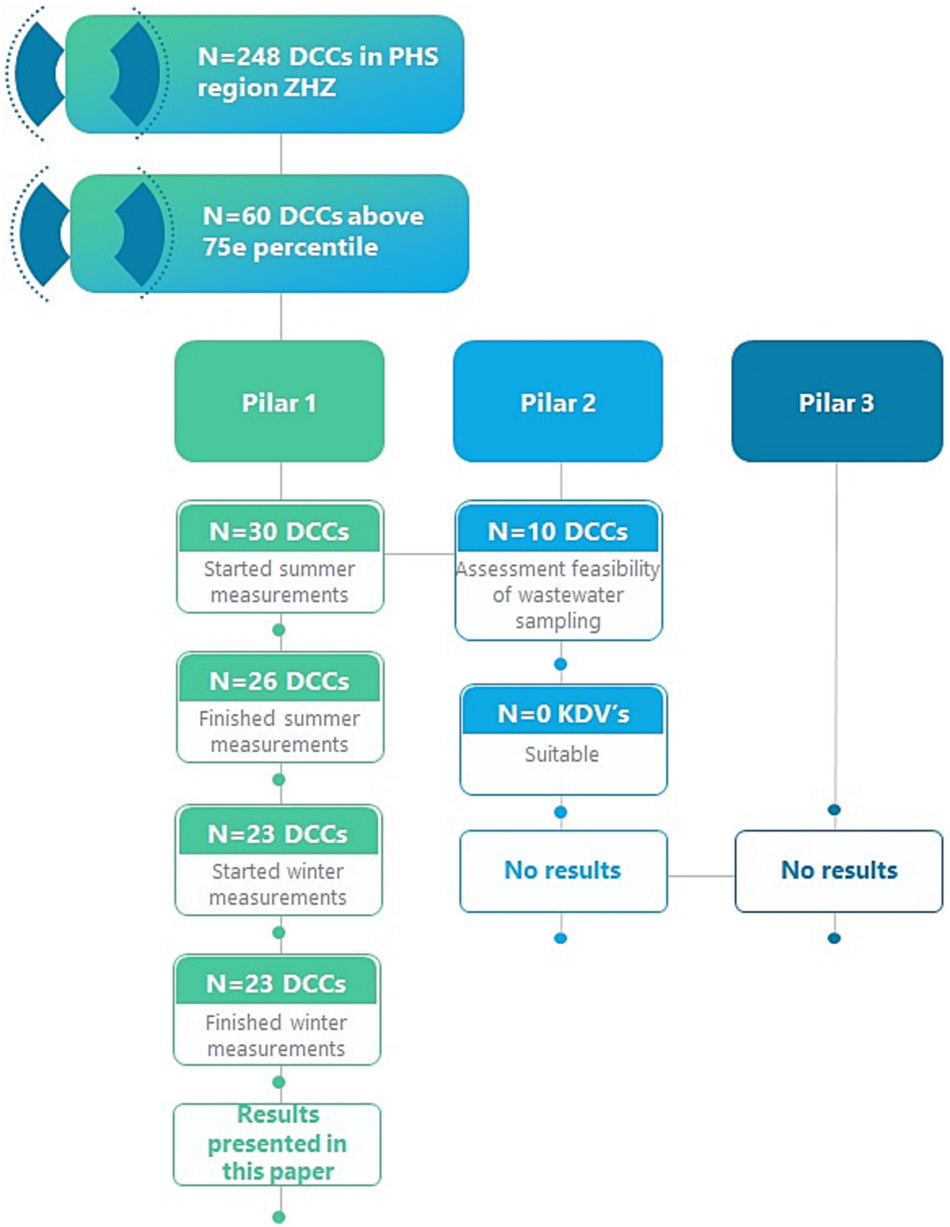
Flowchart of selection, inclusion, and participation of DCCs in the PHS region Zuid-Holland Zuid.

**Figure 2:**
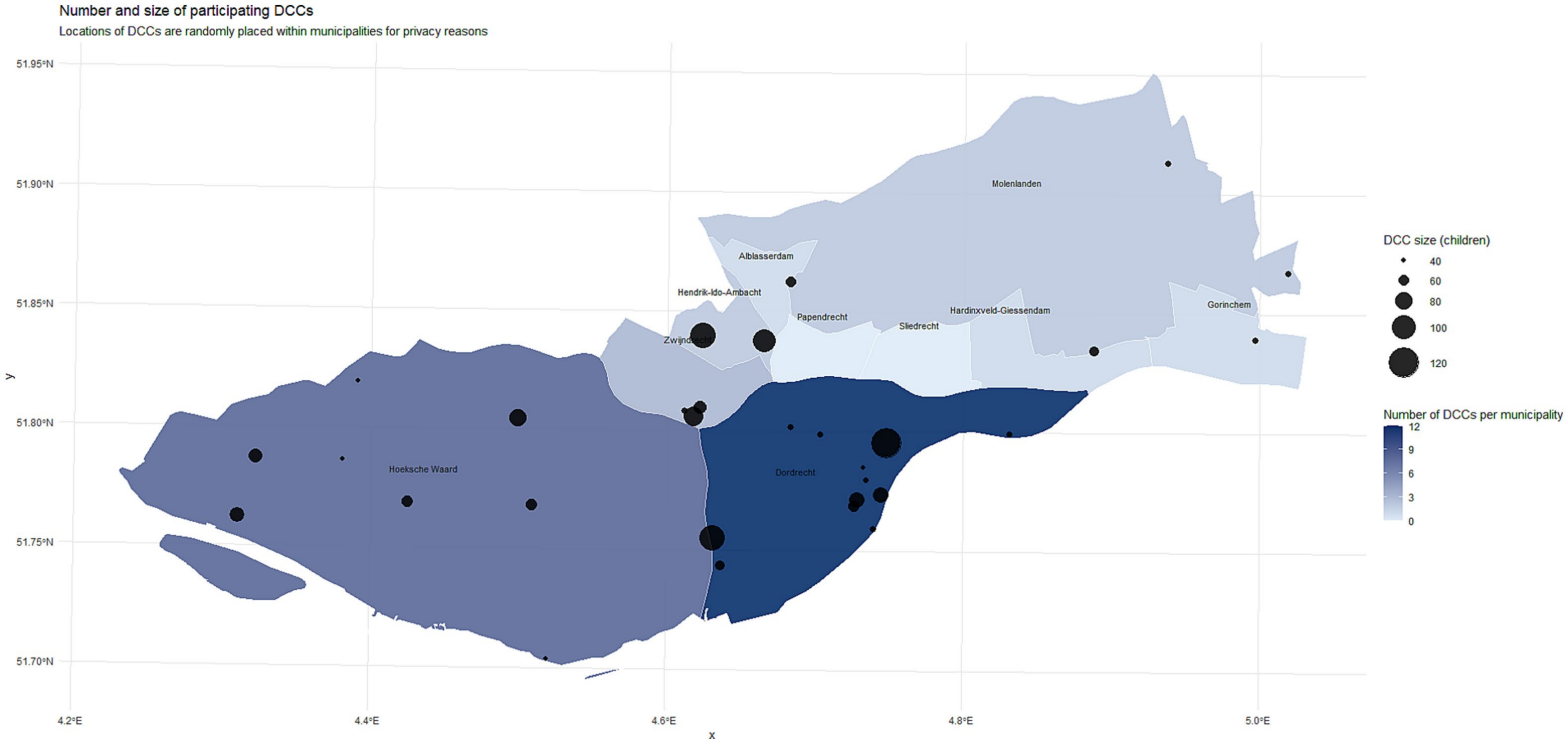
The mapped participating 30 DCCs. The color of the municipality indicates the number of participating DCCs in that municipality; the size of the dot indicates the size of the DCC. See also Supplementary Table 1 for SES scores and urbanization degree of the areas.

In total, 30 of the 60 DCCs that were invited to participate, started collecting data in summer (21 May–12 July), being located in 8 out of 10 municipalities of the PHS region Zuid-Holland Zuid (Figures 1, 2). Twenty six of the initial 30 finished the data collection in summer (2,480 children). Four DCCs dropped out because of staff shortness. Of those 26, 23 DCCs (2,237 children) collected data in winter (21 October–13 December 2024). A DCC enrolled a median of 94.75 children (IQR 66.6–128.6), and a median of 16.25 staff members (IQR 11.3–23.1). DCCs were organized either horizontally (children of similar age grouped together), vertically (children of all ages grouped together) or both.

Participating DCCs were located in neighborhoods with SES scores ranging from −0.38 to 0.31, representing a broadly distributed yet non-extreme socioeconomic profile (SES area scores run from −4 to 4, with 0 representing an average SES). Participating DCCs had a good mix of urbanization degree, ranging from highly urbanized to rural (Supplementary Table 1).

### 3.1 Pillar 1

**Figure 3:**
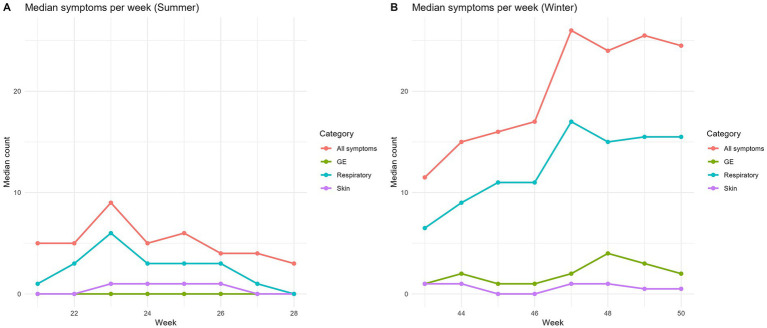
Median number of persons with symptoms per week per DCC in summer **(A)** and winter **(B)**.

**Figure 4:**
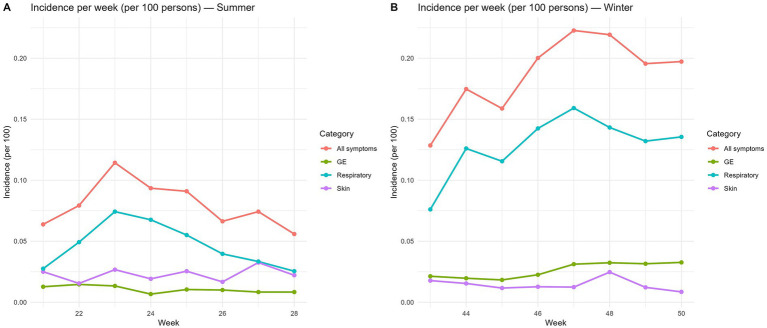
Incidence per 100 persons per week per DCC in summer **(A)** and winter **(B)**.

Tally sheets showed a median of 5 (IQR 0.75–11) symptomatic persons/week/DCC in summer and 20 (IQR 10–31) symptomatic persons/week/DCC in winter. The median of symptomatic persons per week are displayed in Figure 3A (summer) and Figure 3B (winter). The incidences per week in summer are displayed in Figure 4A, and in winter in Figure 4B. The incidence of any kind of symptoms in summer was 8.8/100 persons/week and in winter 20.1/100 persons/week. A Wilcoxon signed-rank test on DCC-level showed that the incidence of persons with any kind of symptoms was statistically significantly higher in winter than in summer (*p* < 0.001). Respiratory infections dominated in both seasons, yet in winter the incidence was statistically significantly higher than in summer (13.9/100 persons/week vs. 5.3/100 persons/week; *p* < 0.001). Incidence of skin infections was slightly higher in summer than in winter but not statistically significant (2.0/100 persons/week vs. 1.7/100 persons/week; *p* = 0.205). Incidence of gastroenteritis symptoms was statistically significantly higher in winter compared to summer (2.5/100 persons/week vs. 1.5/100 persons/week; *p* < 0.001). Although the system was simple and paper-based, most childcare staff still perceived it as burdensome.

### 3.2 Mandatory notifications

Based on the tally sheets of the summer, 18 of the DCCs should have made at least one mandatory notification to the PHS, mostly with regard to skin infections. In fact, the PHS received mandatory notifications from 7 of the participating DCCs—all about skin infections (23%). Among non-participating DCCs (*n* = 218), 8 submitted a mandatory notification during the same period (3.7%). Based on the tally sheets of the winter, 15 of the participating DCCs should have made at least one mandatory notification to the PHS, again almost all with regard to skin infections. In fact, the PHS received mandatory notifications from 4 of the participating DCCs—again all about skin infections (17.4%). Among non-participating DCCs (*n* = 225), 12 submitted a mandatory notification during the same period (5.3%).

### 3.3 Pillar 2

For a total of 10 DCCs the feasibility of wastewater sampling was systematically assessed using maps, site visits, and dye testing. Several site-specific problems were encountered that rendered the wastewater monitoring at those locations impossible. At one DCC, a manhole cover could not be opened and the intended monitoring location not be accessed. Another DCC shared a septic tank with an adjacent cow stable, making isolation of wastewater from the DCC impossible. At another site, the sewer system was heavily polluted and contained a large volume of ‘old’ wastewater, hindering representative sampling of the current situation. At yet another DCC, the dye could not be found anywhere in the sewer system, leading to uncertainty about the actual discharge location of the DCC. For the remaining 6 DCCs, wastewater from the DCC was already mixed with other sources (in the same building and/or from upstream locations) before the wastewater could be accessed for monitoring purposes. As a result of these logistical and infrastructural constraints, wastewater surveillance could not be performed at any of the participating DCCs.

### 3.4 Pillar 3

Since it was not possible to collect wastewater surveillance data, we were not able to make a comparison between data collected in pillar 1 and 2.

Publicly available SARS-CoV-2 wastewater data from the 13 WWTPs serving the study region showed generally low viral loads during both study periods, with limited regional elevations. A modest increase in respiratory symptoms observed in DCCs during week 23 preceded minor increases in regional SARS-CoV-2 levels (17).

## 4 Discussion

The simple paper-based surveillance system implemented in pillar 1 (tally sheets) provided valuable insights in the incidence of infectious diseases at DCCs and revealed illnesses that were largely missed by mandatory notifications, indicating substantial underreporting. Interestingly, the proportion of mandatory notifications was substantially higher among participating DCCs than among non-participating centers during the same periods. Although this study was not designed to evaluate the tally sheets as an intervention, this observation suggests that systematic symptom registration may increase awareness of infectious disease events and encourage reporting. At the same time, participation bias may also have contributed to this difference, as DCCs that are generally more attentive to infectious disease reporting may have been more inclined to participate in the study. This underreporting is likely driven by limited awareness of reporting obligations among childcare staff at the operational level, the perceived limited added value of reporting common outbreaks, the normalization of infections in childcare settings, and competing workload demands of childcare staff (18).

Previous studies, such as the study by Enserink et al. (19), reported gastroenteritis as the most frequently observed syndrome in DCCs, followed by influenza-like illness, particularly during winter months. However, in this study by Enserink, only those illness episodes that resulted in a child being excluded from care were captured. As other research has shown that only approximately 50% of symptomatic children are kept at home (12), such approaches likely underestimate the true burden of disease within these settings. Moreover, most previous studies largely focused on relative risks associated with daycare attendance compared to home care (2, 12), rather than on absolute incidence within daycare populations. Consequently, while broad patterns were anticipated, the expected frequency and distribution of symptoms remained poorly defined. Within this context, our findings were nevertheless surprising. The number of respiratory complaints reported during the summer exceeded expectations based on both statutory notification data and previous literature (2, 12, 19). On the contrary, the incidence of gastroenteritis—particularly in winter—was notably lower than anticipated (1).

To maximize the likelihood of successful wastewater sampling in pillar 2, DCCs were carefully selected based on predefined criteria, including location in stand-alone buildings and not being co-located with schools or after-school care. Nevertheless, we anticipated that infrastructural factors beyond our control might prevent sampling at some of the selected locations. However, the fact that none of the selected sites ultimately proved suitable was unexpected. Our experience suggests that wastewater sampling is more feasible in larger, self-contained facilities such as hospitals or nursing homes, since those facilities frequently have dedicated drainage systems and therefore mixing of wastewater with wastewater from adjacent buildings is less likely. By contrast, DCCs are often embedded in shared or complex infrastructural settings. Feasibility is therefore strongly dependent on the underlying sewer infrastructure.

As an exploratory pilot, this study did not aim to implement comprehensive multi-pathogen wastewater surveillance. Pathogen selection was intentionally restricted to three agents—parvovirus B19, norovirus, and RSV—based on epidemiological relevance in young children, prior evidence of detectability in wastewater, and laboratory feasibility. While this focused approach limits comprehensive etiological coverage, it aligns with the primary objective of assessing operational feasibility rather than establishing pathogen-specific correlations.

Since we could not perform wastewater surveillance, we searched for alternative surveillance methods. We considered for example adapting the sewer system of the individual buildings, as successfully demonstrated in several building-level wastewater surveillance projects. In these studies, researchers installed dedicated sampling points by reopening or constructing manholes at house or dormitory connections, enabling early detection of viral circulation at the level of university campuses and residential buildings (20, 21). However, in our context—DCCs embedded in mixed-use or multi-tenant buildings—these infrastructural modifications were deemed disproportionately demanding in terms of resources and permissions.

Where such direct sampling is infeasible, indirect “block-level” surveillance might still offer potential. Yet this requires extended and frequent monitoring to establish a stable background signal from surrounding households—short-term datasets are vulnerable to variability and noise (e.g., spatial/temporal variance in sewage signals (22)) and may fail in low-incidence settings or small population sizes (23, 24). Moreover, Enserink et al. (19) stress that infectious disease incidence in childcare settings varies significantly between years, and that short-term data may misrepresent true background levels. Consequently, our current study design does not allow for reliable differentiation between incidental peaks and meaningful deviations from baseline, limiting the interpretability of potential wastewater signals—whether direct or indirect. Although regional SARS-CoV-2 wastewater data were descriptively reviewed, the highly aggregated and heterogeneous catchment areas precluded meaningful interpretation of DCC-level syndromic trends in relation to community-level signals.

We also considered implementing diaper-based stool sampling, as this method has been successfully applied in previous DCC surveillance studies (25–30). While such sampling requires additional effort from DCC staff or parents and raises consent-related considerations, initial responses from DCC staff were generally positive. Several staff members expressed no objections and indicated that the procedure would not pose any additional burden, others expressed uncertainty regarding its permissibility, still others preferred not to participate but felt a responsibility to contribute, having already agreed to the symptom registration on the tally lists. We proposed that the diaper sampling would have followed an opt-out procedure, with parents being informed via the digital parent portal. Samples would have been pooled and anonymized, precluding any traceability to individual children—methodologically comparable to wastewater surveillance. However, the METC-in this case-required active informed consent from each parent and did not approve our proposed opt-out procedure. This requirement introduced significant practical and ethical challenges. To meet the informed consent requirement, DCCs would have had to contact all parents to request permission to share their contact details with the research team. Subsequently, the researchers would have had to individually contact the parents of 2,237 children to provide study information, allow for a reflection period, and register their informed consent decision. This would not only have created a substantial administrative burden, but also contradicted the study’s design principle of collecting only aggregated, non-identifiable data. For these reasons, the diaper sampling component was ultimately not implemented.

### 4.1 Strengths and limitations

Our study has some limitations. First, due to reasons described above, no diagnostic testing was performed, preventing confirmation of syndromic reports. Syndromic surveillance based on predefined symptoms inherently carries some risk of misclassification. Although symptoms were recorded when considered of suspected infectious origin in line with statutory reporting criteria, non-infectious causes cannot be fully excluded. However, the objective of Pillar 1 was to capture infectious signals at symptom level rather than laboratory-confirmed etiologies. Second, the syndromic surveillance system was based on weekly tally sheets rather than daily resolution. Although this choice reflected feasibility concerns raised by consulted childcare staff during the development of the pilot, it precluded the calculation of corrected incidence measures as described by Enserink et al. (19), which require day-level data. Third, although symptom categories were based on statutory reporting criteria, the absence of additional training may have resulted in inter-observer variability between staff members. Fourth, staff and non-diaper-wearing children were not registered separately. While appropriate from a wastewater-oriented design perspective, this precluded separate epidemiological analyses by age group and may have obscured differences in clinical presentation. Fifth, the monitoring period was relatively short (two periods of 8 weeks), restricting the ability to assess temporal trends or seasonal patterns robustly. Finally, we deliberately included only larger DCCs, however, smaller DCCs may exhibit different transmission dynamics, limiting the generalizability of our results.

Our study also has notable strengths. We included a substantial number of children, with approximately half of all invited DCCs agreeing to participate. Moreover, participating DCCs were from nearly all 10 municipalities in our PHS region, providing a heterogeneous sample representing both urban and rural contexts and a broad range of socioeconomic backgrounds. This diversity makes the region broadly representative of the wider Dutch context, supporting the generalizability of our findings to other regions in the Netherlands.

### 4.2 Conclusion

This study addressed the need for more reliable incidence data in childcare settings by piloting a syndromic surveillance system based on weekly tally sheets. Although this approach yielded meaningful epidemiological insights, it was perceived as administratively demanding by childcare staff, indicating that this method is not a sustainable surveillance system. Wastewater surveillance, while conceptually promising, proved less feasible than anticipated under the operational conditions of this study. While broad implementation of wastewater surveillance in DCCs appears challenging, future research could explore whether a sentinel-based approach in carefully selected, infrastructurally suitable centers may be feasible. Sentinel surveillance models have proven effective in other public health contexts; however, whether such a model would provide meaningful and sustainable signals in the DCC setting remains uncertain and requires empirical validation. Diaper sampling was considered as it offered additional diagnostic value, yet raised practical and ethical concerns. Collectively, these findings highlight the challenges of implementing sustainable surveillance in non-medical community settings. Future efforts should prioritize the development of low-burden, scalable, and methodologically robust surveillance systems that operate independently of healthcare-seeking, testing, and reporting behaviors—either or not in sentinel centers. Such systems could substantially strengthen infectious disease surveillance in DCCs by enabling more accurate estimation of background prevalence and earlier detection of infection trends, thereby facilitating timelier and more targeted public health responses.

## Data Availability

The raw data supporting the conclusions of this article will be made available by the authors, without undue reservation.
