# Supplementary material for: Improving surveillance of infectious diseases in child daycare settings: insights from a pilot study in the Netherlands
Source: Front Public Health. 2026 Apr 10;14:1797334. doi: 10.3389/fpubh.2026.1797334 (PMC13106512; doi:10.3389/fpubh.2026.1797334)
Supplement: Supplementary file 1 [file Table_1.DOCX]

Supplementary Material

# Supplementary Figures and Tables

Supplementary Table 1: DCC characteristics

| **Municipality** | **Socio-economic status** | **Urbanisation degree** | | | | |
| --- | --- | --- | --- | --- | --- | --- |
|  |  | **1** | **2** | **3** | **4** | **5** |
| Alblasserdam | -0.021 |  | 1 |  |  |  |
| Dordrecht | -0.377 to 0.245 | 7 | 4 | 1 |  |  |
| Gorinchem | -0.002 |  | 1 |  |  |  |
| Hardinxveld-Giessendam | 0.125 |  |  | 1 |  |  |
| Hendrik-Ido-Ambacht | 0.105 to 0.208 |  | 2 |  |  |  |
| Hoeksche Waard | 0.098 to 0.314 |  | 2 | 2 | 2 | 2 |
| Molenlanden | 0.262 to 0.296 |  |  |  |  | 2 |
| Zwijndrecht | -0.102 to -0.086 | 2 | 1 |  |  |  |

Urbanisation degree categorized by number of addresses per km2 as 1) highly urbanized (>2.500), 2) urbanized (1.500–2.000), 3) moderately urbanized (1.000–1.500), 4) low urbanized (500–1.000) and 5) rural (<500).

Socioeconomic status (SES) expressed as a normalized score (-4 to +4) based on level of income, employment and educational level per postal code area. A high score indicates a low SES of that postal code area. The score was constructed using multiple correspondence analysis (MCA) and normalized to have a mean of 0 across the Dutch population. Higher values indicate lower SES.
